# Pyrimethamine Triggers the Apoptotic Pathway in Mucoepidermoid Carcinoma in Cell‐Based Models

**DOI:** 10.1111/jop.70024

**Published:** 2025-08-06

**Authors:** Hyun‐Ji Kim, Dong‐Guk Park, Su‐Jung Choi, Jae‐Jin Cho, Seong‐Doo Hong, Sung‐Dae Cho

**Affiliations:** ^1^ Department of Oral Pathology School of Dentistry and Dental Research Institute, Seoul National University Seoul Republic of Korea; ^2^ Department of Dental Regenerative Biotechnology, School of Dentistry Seoul National University Seoul Republic of Korea

**Keywords:** apoptosis, caspase 8, mcl‐1, mucoepidermoid carcinoma, pyrimethamine

## Abstract

**Background:**

Mucoepidermoid carcinoma (MEC) is the most prevalent salivary gland malignancy, with a poor prognosis in high‐grade tumors at diagnosis. This highlights the need for effective antitumor agents for treating MEC. Therefore, we aimed to investigate the antineoplastic efficacy of pyrimethamine (PYR), a Food and Drug Administration‐approved antiparasitic medicine, to repurpose it as an alternative therapeutic option for treating human MEC.

**Methods:**

The trypan blue exclusion assay, cell counting kit‐8 assay, and live/dead assay were performed to assess the antiproliferative efficacy of PYR. PYR‐induced apoptosis was confirmed with 4′,6‐diamidino‐2‐phenylindole staining, cell cycle analysis, and annexin V/propidium iodide staining. A western blot assay was conducted to measure changes in cleaved caspase 8 and myeloid cell leukemia‐1 (Mcl‐1) expression after PYR treatment. Mcl‐1 overexpression was used to further confirm the apoptosis‐inducing activity of PYR. The hanging drop method was employed to assess the efficacy of PYR in a three‐dimensional culture system.

**Results:**

PYR‐induced apoptotic cell death in the YD‐15 high‐grade MEC cell line by promoting apoptosis, as evidenced by MCl‐1 proteasomal degradation and increased cleaved caspase 8 expression.

**Conclusion:**

Our results indicate that PYR can effectively target human MEC by inducing both intrinsic and extrinsic apoptotic pathways.

## 1 Introduction

The incidence of salivary gland tumors was estimated at 55 003 annually in 2022 based on the report released by the International Agency for Research on Cancer in 2024 [1], with an expected increase of over 65% by 2050 [2]. Mucoepidermoid carcinoma (MEC) is the most prevalent salivary gland malignancy, predominantly located in the parotid gland, and is characterized by a combination of distinct cell types, including epidermoid, mucous, and intermediate cells [3]. The clinical prognosis for low‐ and intermediate‐grade MEC is generally favorable, with a 5‐year survival rate approaching 95%; however, the rate drops to approximately 50% in high‐grade MEC [4]. Further, recurrence and distant metastasis persist even decades after the initial treatment, particularly, in cases associated with high T‐stage or lymph node involvement [5, 6]. Surgical dissection, frequently accompanied by adjuvant radiotherapy, has been the primary treatment for MEC [7] because of the lack of effective nonsurgical therapeutic alternatives for treating MEC and preventing recurrence, particularly, in advanced disease [6].

Pyrimethamine (PYR) is a Food and Drug Administration (FDA)‐approved antiparasitic drug administered to combat toxoplasmosis and malaria [8]. The relatively recent era of oncology research has imparted tremendous efforts to discover therapeutic alternatives aimed at reducing the devastating side effects and resistance associated with conventional chemotherapeutic drugs. Building on this momentum, several studies have substantiated the potential of PYR as an antineoplastic agent in various human cancers such as breast cancer, leukemia, lung cancer, and melanoma [9]. Of importance, PYR restricts the DNA production of cancer cells by impeding the interaction between folic acid and dihydrofolate reductase (DHFR), as it does in protozoa [10, 11]. Further, it employs a wide range of mechanisms to hinder multiple tumor progression in humans [12, 13, 14]. However, to the best of our knowledge, few studies have investigated the antitumorigenic activity of PYR in human MEC.

In the present study, we aimed to assess the potential of PYR as a therapeutic alternative for treating MEC. We revealed that PYR reduces the expression of myeloid cell leukemia‐1 (Mcl‐1) through posttranslational regulation and stimulates the activity of caspase 8, resulting in the cleavage of caspase 3 and ultimately inducing apoptosis‐associated cellular downfall in both two‐dimensional (2D) and three‐dimensional (3D) cell culture models.

## 2 Materials and Methods

### 2.1 Cell Culture and Reagents

The YD‐15 human MEC cell line was obtained from Yonsei University (Seoul, Republic of Korea) and cultured in RPMI 1640 medium (WELGENE, Gyeongsan, Republic of Korea) supplemented with 10% fetal bovine serum and 1% penicillin/streptomycin. Cells were incubated at 37°C in a humidified atmosphere with 5% CO_2_. Experiments began when the cells reached around 50% confluency. Dimethyl sulfoxide (DMSO) was used as a solvent for all chemicals, which were stored at −20°C. The cells were divided into two groups: (i) DMSO‐treated control and (ii) PYR (MedChemExpress, Monmouth Junction, NJ, USA)‐treated at 15 and 30 μM for 2D culture, and 120 μM for 3D culture.

### 2.2 Western Blot Analysis

Whole‐cell proteins were lysed using RIPA buffer (Millipore, Burlington, MA, USA) diluted to 1× concentration, containing phosphatase (Thermo Scientific, Waltham, MA, USA) and protease inhibitors (Roche, Basel, Switzerland). Protein concentration was measured with a DC Protein Assay Kit (BIO‐RAD, Madison, WI, USA). Equal protein amounts were separated by sodium dodecyl sulfate‐polyacrylamide gel electrophoresis and transferred to a polyvinylidene difluoride membrane. The membrane was blocked with 5% skim milk in TBST for 1 h at room temperature (RT), followed by incubation with the primary antibody for 6 h at RT. After washing, the membrane was treated with HRP‐conjugated secondary antibodies for 3 h at RT. Protein expression was detected using WestGlow FEMTO Chemiluminescent Substrate (BIOMAX, Seoul, Republic of Korea) on x‐ray film. The list of antibodies, including source and dilution, is provided in Table S1.

### 2.3 Quantitative PCR

RNA was extracted with TRIzol reagent (Life Technologies, Carlsbad, CA, USA), and 1 μg of RNA was reverse transcribed into cDNA using the AMPIGENE cDNA Synthesis Kit (Enzo Life Sciences, USA). The cDNA was then amplified via PCR using AMPIGENE qPCR Green Mix Hi‐Rox (Enzo Life Sciences, Farmingdale, NY, USA). Real‐time PCR was conducted on the Applied Biosystems StepOne Plus Real‐Time PCR System (Applied Biosystems, Foster City, CA, USA) under the following conditions: an initial 95°C for 2 min, followed by 40 cycles of 95°C for 10 s and 60°C for 30 s. GAPDH expression was used to normalize the relative amount of each gene, as determined by the 2^−ΔΔ^ *C*_t_ method. The qPCR primers used were: Mcl‐1 sense: 5′‐GTA TCA CAG ACG TTC TCG TAA GG‐3′, Mcl‐1 antisense: 5′‐CCA CCT TCT AGG TCC TCT ACA T‐3′; GAPDH sense, 5′‐GTG GTC TCC TCT GAC TTC AAC‐3′; GAPDH antisense, 5′‐CCT GTT GCT GTA GCC AAA TTC‐3′.

### 2.4 Mcl‐1 Transient Overexpression

The previously constructed pcDNA3.1‐Mcl‐1 overexpression vector was used [15]. The YD‐15 cell was transfected with either 250 ng blank pcDNA3.1 vector or the pcDNA3.1‐Mcl‐1 vector using Lipofectamine 2000 (Thermo Scientific, Waltham, MA, USA) for 12 h according to the manufacturer's guidelines.

### 2.5 Spheroid Formation

Cell spheroids were generated using the hanging drop aggregation method, as described in a previously published protocol [16]. Briefly, 20 μL of 2.5 × 10^5^ cells/ml, diluted in 0.24% methylcellulose RPMI, was dropped onto the lid of a 100 mm dish. The lid was then placed upside down onto a plate filled with 8 mL phosphate‐buffered saline to prevent drying out. After 48 h, the spheroids were transferred into 96‐well 0.5% Poly(2‐hydroxyethyl methacrylate)‐coated plates for further analysis.

### 2.6 Statistical Analysis

Graphs were created using GraphPad Prism version 8.0 (GraphPad Software, San Diego, CA, USA), while statistical analyses were performed with SPSS version 26.0 (SPSS, Chicago, IL, USA). Data from three separate biological experiments are presented as mean ± standard deviation (SD). To determine statistical significance, appropriate tests were selected based on data distribution and variance. Normality was assessed using the Shapiro–Wilk test, and homogeneity of variances using Levene's test. Depending on the results, either a two‐tailed Student's *t* test or one‐way ANOVA with Bonferroni or Dunnett's T3 post hoc test was applied. For nonnormally distributed data, the Kruskal–Wallis test followed by pairwise Mann–Whitney *U* tests was used. Results were considered significant if the *p*‐value was below 0.05 (* or #).

## 3 Results

### 3.1 PYR Induces Caspase‐Dependent Extrinsic Apoptotic Cell Death in the YD‐15 Cell Line

**FIGURE 1:**
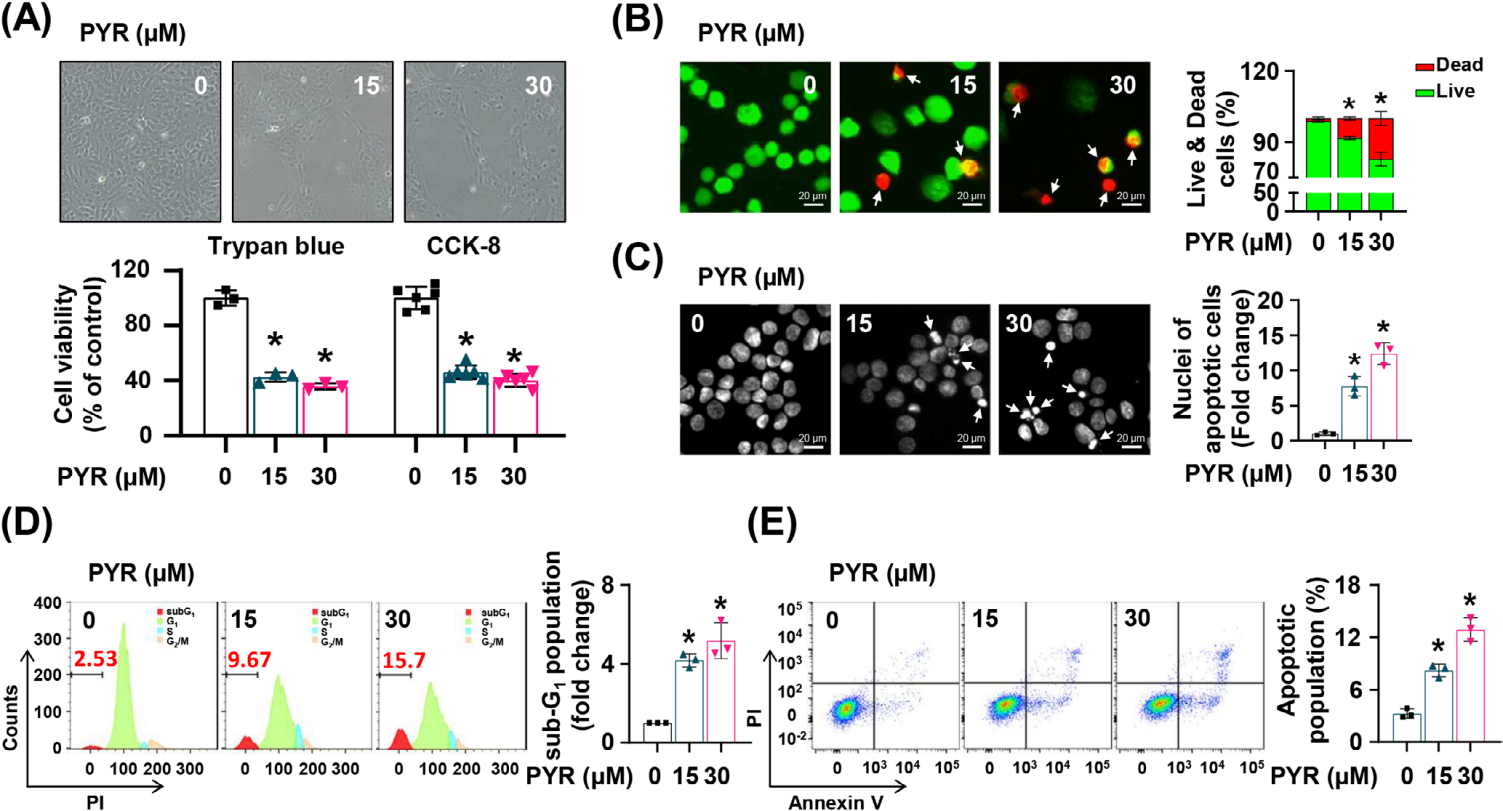
The effect of PYR on proliferation and apoptosis in the YD‐15 cell line. YD‐15 was exposed to DMSO or the designated concentrations of PYR for 48 h. (A) The upper panel displays representative images of cells treated with either DMSO or PYR. Cell viability was assessed in the lower panel using both the trypan blue exclusion method and the CCK‐8 assay. (B) The images show live cells stained with Calcein AM (green) and dead cells stained with EthD‐1 (red). White arrows indicate dead cells. Magnification, ×400; Scale bar, 20 μm. (C) Representative images of DAPI‐stained cells. White arrows indicate apoptotic nuclei. Magnification, ×400; scale bar, 20 μm. (D) Flow cytometry analysis was used to estimate the sub‐G1 population in cells treated with PYR. (E) The apoptotic cells were determined by flow cytometry analysis. All graphs represent the mean ± SD from three independent experiments. Statistical significance is denoted by * (*p* < 0.05), determined by one‐way ANOVA.

**FIGURE 2:**
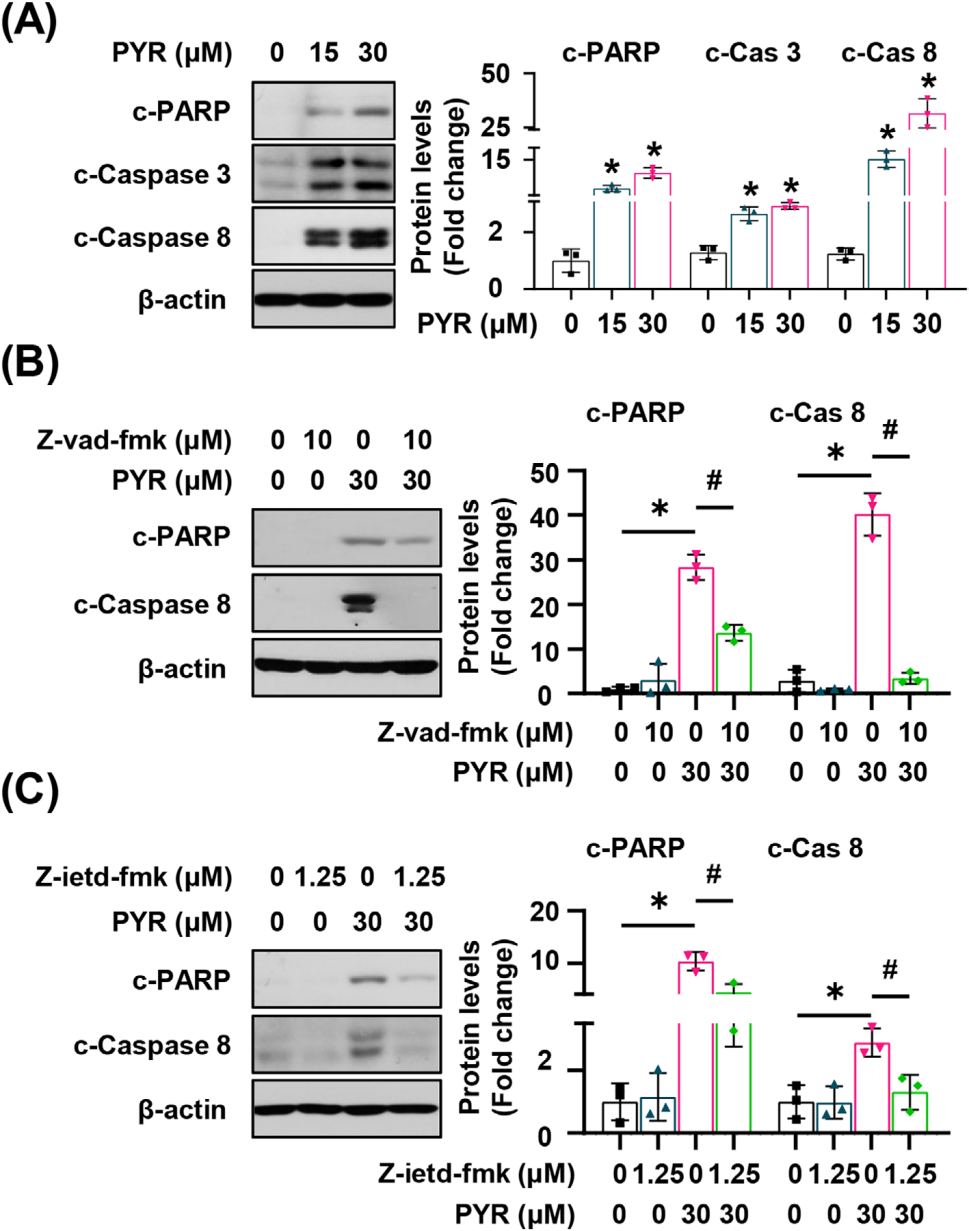
The effect of PYR on caspase‐dependent apoptosis in the YD‐15 cell line. YD‐15 cell was exposed to DMSO or the indicated concentrations of PYR for 48 h. (A) The western blot images illustrate the expression levels of c‐PARP, c‐Caspase 3, and c‐Caspase 8. (B and C) YD‐15 cell was preexposed to DMSO or the indicated concentration of Z‐vad‐fmk or Z‐ietd‐fmk 2 h before treatment of PYR for 48 h. The images show the expression of c‐PARP and c‐Caspase 8, with β‐Actin serving as the loading control. Three independent experiments were conducted to represent all the graphs with mean ± SD. * or #, *p* < 0.05 by one‐way ANOVA.

To investigate the anticancer effect of PYR on MEC, we performed the trypan blue assay and cell counting kit assay on the YD‐15 cell line after PYR treatment for 48 h. PYR notably attenuated the cell viability in a concentration‐dependent manner (Figure 1A). Further, we observed an increase in the dead cell fraction, which was stained red in the PYR‐treated cells (Figure 1B). To identify the mechanism of cell death induced by PYR, 4′,6‐diamidino‐2‐phenylindole staining was performed. Apoptotic nuclei, characterized by nuclear fragmentation and chromatin condensation, were readily detected in the PYR‐treated groups (Figure 1C). Subsequently, two flow cytometry methods, the sub‐*G*_1_ assay and annexin V/PI staining, were employed to further confirm apoptotic cell death induction by PYR in the YD‐15 cell line. These results revealed an increase in the sub‐G_1_ population and annexin V‐positive cell population, respectively (Figure 1D,E). After PYR treatment, the expression of cleaved caspase 8, caspase 3, and poly (ADP‐ribose) polymerase (PARP) was remarkably increased (Figure 2A). This indicates that PYR stimulated caspase‐mediated extrinsic apoptosis in the YD‐15 cell line, as supported by the increased DR5 protein expression after PYR treatment (Figure S1). To validate this, we treated the cells with Z‐vad‐fmk, a pan‐caspase inhibitor, for 2 h before PYR treatment. Pretreatment with Z‐vad‐fmk significantly downregulated cleaved caspase 8 and PARP (Figure 2B). Additionally, the anti‐apoptotic efficacy of the caspase 8‐specific inhibitor, Z‐ietd‐fmk, was tested, and we obtained similar results as before (Figure 2C). Collectively, these findings indicate that PYR attenuates the growth of the YD‐15 cell line, which is caused by caspase‐dependent extrinsic apoptotic cell death.

### 3.2 PYR Downregulates Mcl‐1 Protein Expression by Proteasome‐Dependent Degradation

**FIGURE 3:**
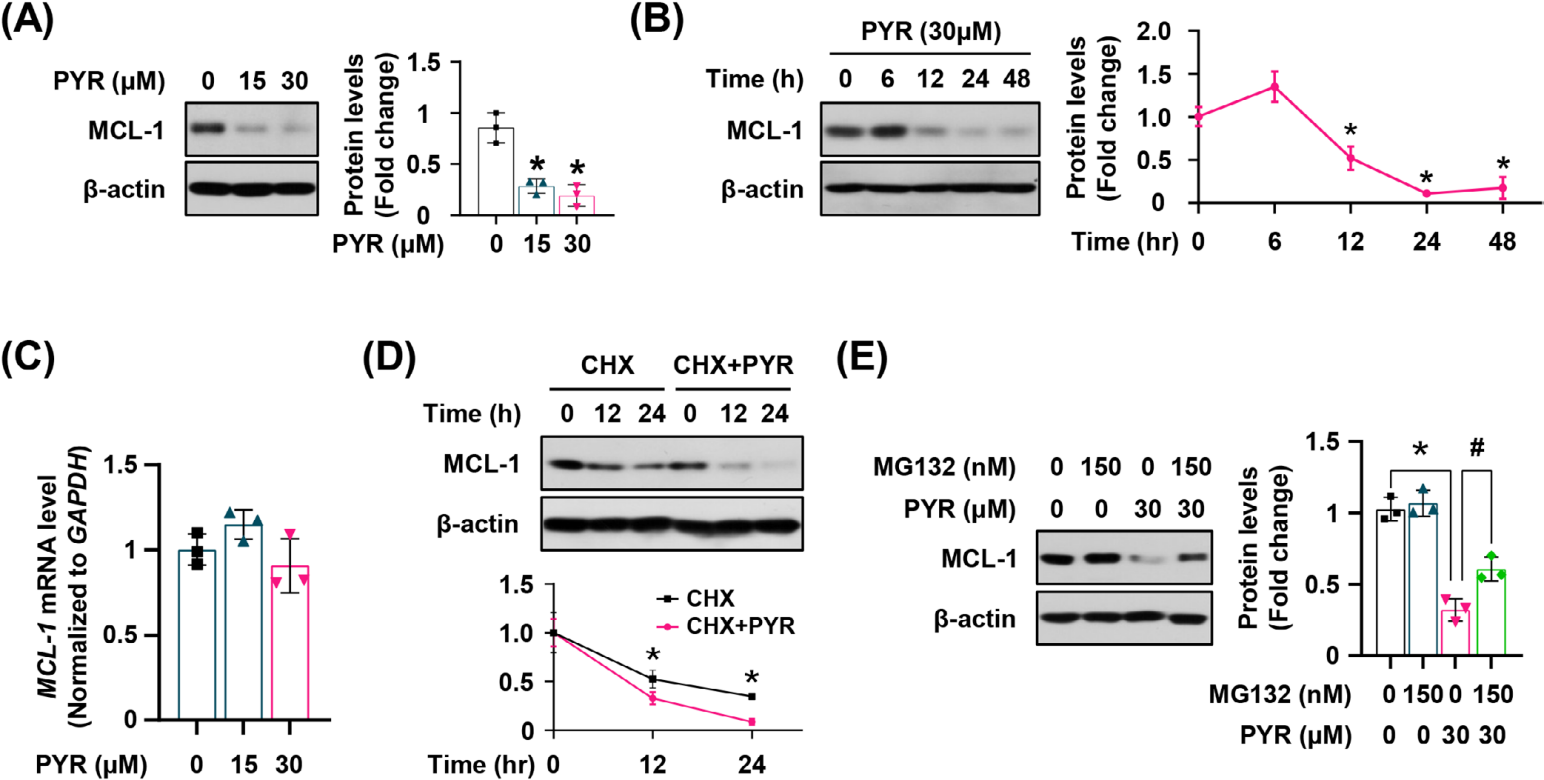
The effect of PYR on the stability of Mcl‐1 in the YD‐15 cell line. YD‐15 cell was exposed to DMSO or the indicated concentrations of PYR for 48 h or the indicated time. (A, B) The image shows the expression of Mcl‐1. (C) qRT‐PCR was performed to evaluate Mcl‐1 mRNA expression. (D, E) Either CHX or MG142 was treated 2 h before PYR treatment for the indicated time point (CHX) or 48 h (MG132). Representative images show the expression of Mcl‐1. β‐Actin serves as the loading control. All graphs represent the mean ± SD of three independent experiments. * or #, *p* < 0.05 by one‐way ANOVA.

We aimed to determine the mediator of apoptotic cell death in the YD‐15 cell line induced by PYR and screened the expression of representative pro‐ and anti‐apoptotic proteins that fluctuated after PYR treatment (Figure S2). Thus, we revealed that PYR significantly reduced Mcl‐1, a powerful antiapoptotic protein associated with intrinsic apoptosis, in a concentration‐ and time‐dependent manner (Figure 3A,B). This indicates the involvement of intrinsic apoptosis as well. We aimed to further investigate the mechanism by which Mcl‐1 expression is regulated by PYR. The previous study demonstrated that PYR could affect the Mcl‐1 mRNA level by regulating signal transducer and activator of transcription 3 (STAT3), an upstream transcriptional factor of Mcl‐1; thus, we verified the alteration in Mcl‐1 mRNA expression. However, mRNA levels remained relatively stable in the PYR‐treated group compared with the control group (Figure 3C). Therefore, we shifted our focus to the post‐translational regulation of Mcl‐1, as it is well‐known for its rapid turnover through various regulatory mechanisms [17]. PYR administration considerably compromised the stability of Mcl‐1 when new protein synthesis was blocked by CHX, a protein synthesis inhibitor, relative to the control (Figure 3D). Reduced Mcl‐1 protein expression was readily recovered upon MG132 treatment, indicating the involvement of proteasomal degradation in its downregulation (Figure 3E). Taken together, we revealed that Mcl‐1 regulation by PYR is associated with posttranslational regulation, particularly, through proteasomal degradation, and is also associated with intrinsic apoptosis induction in the YD‐15 cell line.

### 3.3 Mcl‐1 Is the Pivotal Mediator of PYR‐Stimulated Apoptosis in the YD‐15 Cell Line

**FIGURE 4:**
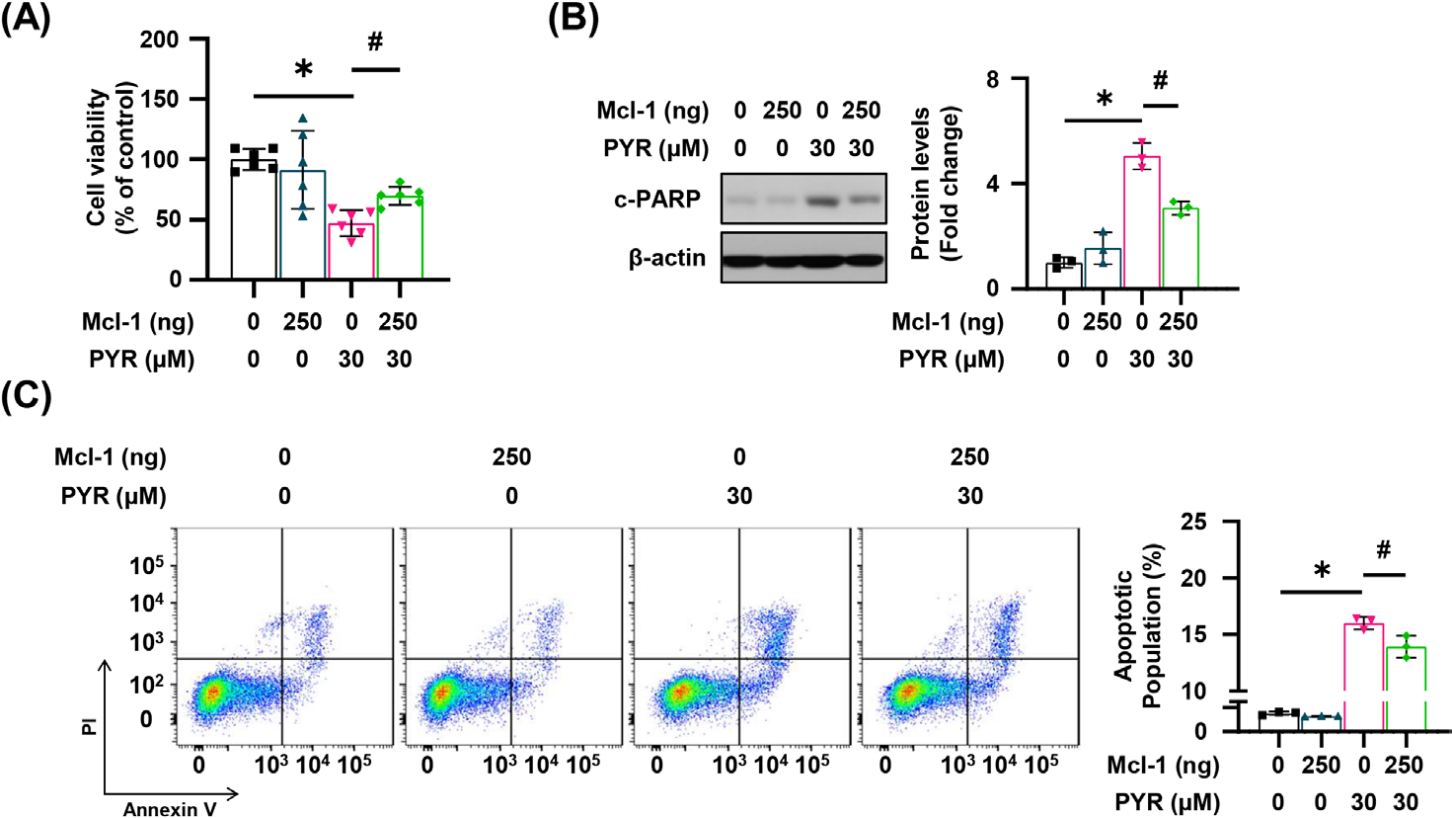
The effect of Mcl‐1 overexpression on the PYR‐mediated apoptotic cell death in the YD‐15 cell line. YD‐15 cell was transfected with blank pcDNA3.1 vector or the pcDNA3.1‐Mcl‐1 vector for 12 h and administered with PYR for 48 h. (A) Trypan blue exclusion assay was utilized to determine cell viability. (B) The western blot images show the expression of c‐PARP with β‐actin used as a loading control. (C) The Annexin V‐positive populations were evaluated by flow cytometry analysis. Three independent experiments were conducted to represent all the graphs with mean ± SD. * or #, *p* < 0.05 by one‐way ANOVA.

To assess the importance of Mcl‐1 in PYR‐mediated apoptosis, we investigated whether Mcl‐1 recovery could mitigate apoptotic cell death (Figure S3). As expected, PYR‐induced cell death was mitigated in the Mcl‐1 overexpressing cells (Figure 4A). Further, the cleavage of PARP was attenuated, and the apoptotic population was reduced by Mcl‐1 overexpression (Figure 4B,C). Consequently, these data indicate that PYR induces cell death through intrinsic apoptosis, in which Mcl‐1 serves, at least in part, as a salient mediator in the YD‐15 cell line.

### 3.4 PYR Demonstrates Effective Antitumorigenic Activity in the 3D Spheroid Culture Model

**FIGURE 5:**
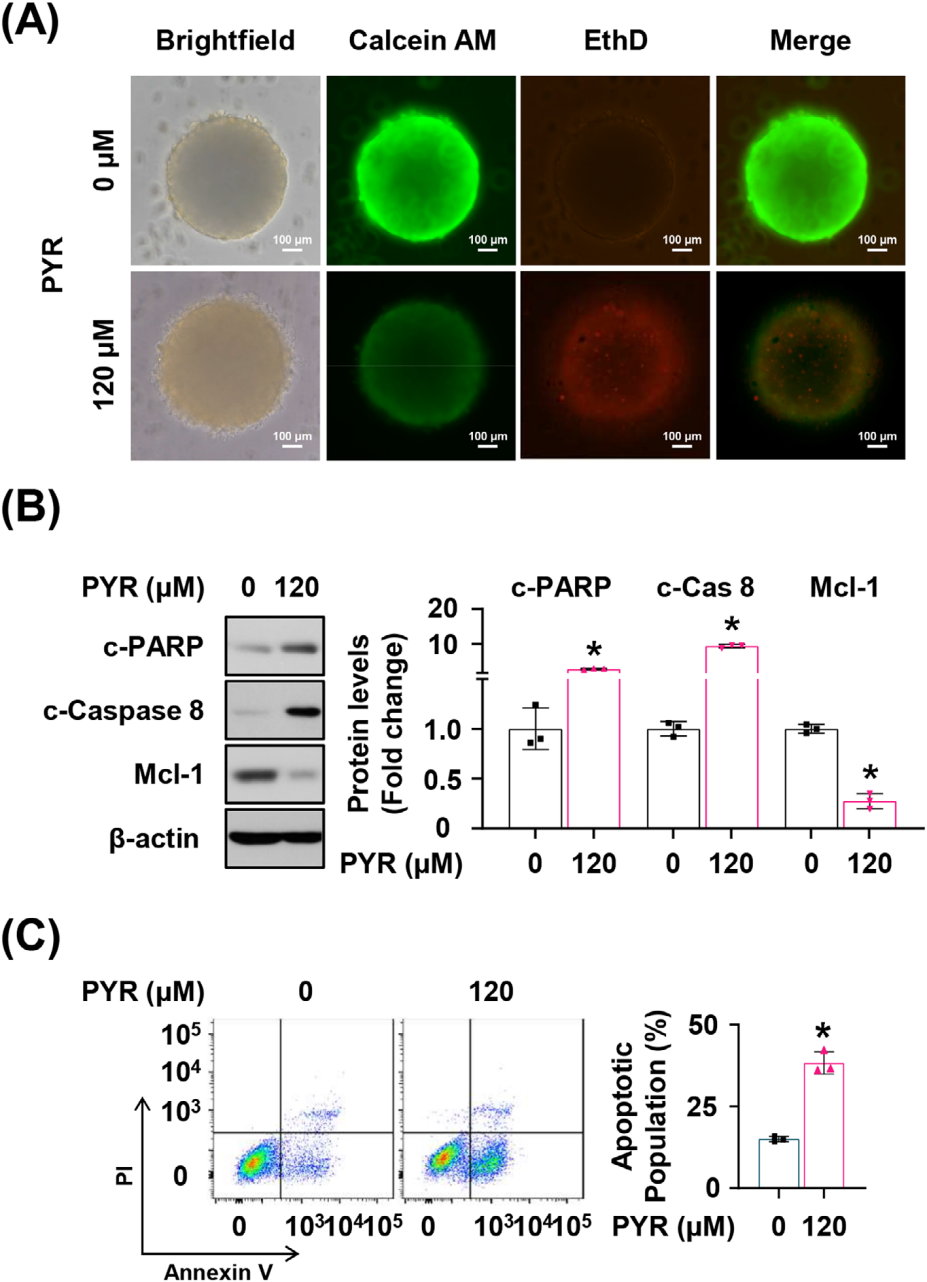
The effect of PYR on the 3D spheroid of YD‐15 cell. YD‐15 cell spheroids were exposed to DMSO or the indicated concentrations of PYR for 48 h. (A) The images show live cells stained with Calcein AM (green) and dead cells stained with EthD‐1 (red). Magnification, ×40; Scale bar, 100 μm. (B) Representative western blot images show the expression of c‐PARP, c‐caspase 8, and Mcl‐1, with β‐actin used as a loading control. (C) The Annexin V‐positive populations were evaluated by flow cytometry analysis. All graphs represent the mean ± SD from three independent experiments. Statistical significance is denoted by * (*p* < 0.05), determined using a two‐tailed Student's *t* test.

To assess the feasibility of PYR in preclinical trials, we fabricated the tumor spheroids employing the hanging drop method and selected 120 μM as an appropriate PYR concentration in the 3D culture system, which induced remarkable cell death (Figure S4). Treatment resulted in noticeable morphological changes in the peripheral cells of the cancer spheroids, displaying a more disorganized and fragmented structure (Figure 5A). Additionally, the proportion of dead cells relative to live cells was significantly increased compared with the control spheroids, as evidenced by the decrease in green fluorescence and the increase in red fluorescence. To identify whether PYR induces cell death via the same mechanism in the 3D spheroid model as observed in the 2D cell culture, we analyzed the expression of three key proteins. Similar to 2D results, despite a difference in the treatment concentration, cleaved caspase 8 and PARP expression markedly increased, whereas Mcl‐1 expression decreased upon PYR administration, indicating that PYR may induce Mcl‐1‐mediated and caspase‐dependent apoptosis in cancer cell spheroids (Figure 5B). This was further supported by the increase in apoptotic populations in the PYR‐treated spheroids compared with the control spheroids (Figure 5C). Consequently, PYR demonstrated effective antitumor activity in the 3D spheroid cell culture model, consistent with its effects observed in the 2D cell culture model.

## 4 Discussion

In this study, we investigated the effectiveness of the antiparasitic drug PYR in treating MEC by simultaneously stimulating caspase 8‐dependent extrinsic apoptosis and Mcl‐1 reduction‐mediated intrinsic apoptosis in both 2D and 3D cell culture models.

Apoptosis has two strongholds, the intrinsic and extrinsic pathway, which have different soldiers and tactics. In the intrinsic pathway, cellular stress is the main inducer and mitochondria act as a general initiator, involving caspase 9 and other Bcl‐2 family proteins, including Mcl‐1. Conversely, in the extrinsic pathway, extracellular signals serve as the commanders, activating the death receptors, which then deploy caspase 8 as their chief executioner, finally resulting in cellular downfall [18]. In the current study, PYR‐induced apoptosis involved caspase activation, particularly caspase 8, and also caused downregulation of Mcl‐1. Based on the results obtained using a pan‐caspase inhibitor and a caspase 8 specific inhibitor, where PARP cleavage, an irreversible hallmark of apoptosis, was blocked (Figure 2B,C), we speculated that the PYR‐induced cell death may rely on extrinsic caspases activations, particularly, caspase 8. Meanwhile, Mcl‐1 protein stability was compromised following PYR administration (Figure 3), suggesting that intrinsic apoptotic signaling is likewise associated with the antiproliferative effect of PYR. This was evidenced by Figure 4, which shows partial rescue of PYR‐mediated apoptosis due to aberrant Mcl‐1 expression before PYR treatment. In line with the concurrent activation of both endogenous and exogenous apoptotic pathways after anticancer agent treatment observed in previous studies [19, 20], PYR appears to engage both apoptosis pathways in exerting its anticancer effect.

Although PYR induced both intrinsic and extrinsic apoptosis pathways, involving Mcl‐1 and cleaved caspase 8, the caspase 8‐specific inhibitor Z‐ietd‐fmk also downregulated c‐PARP expression, indicating the crucial role of caspase 8 activity in apoptosis induction. In Figure S2A, tBID expression tended to increase upon PYR treatment. Since tBID acts as a mediator between extrinsic and intrinsic apoptosis [21], we assumed that extrinsic apoptosis is initiated first, followed by subsequent intrinsic apoptosis. However, the precise interconnection between the intrinsic and extrinsic apoptotic pathways prompted by PYR in MEC remains to be elucidated.

PYR is a well‐known DHFR inhibitor, an enzyme essential for the production of folic acid, a cofactor required for DNA synthesis. Additionally, PYR has recently been recognized as a STAT3 inhibitor [22]. STAT3 is a pivotal transcriptional activator of a myriad of downstream target genes, including Mcl‐1 [23]. An earlier study revealed that reduced STAT3 transcriptional activity due to PYR‐induced folate metabolism deficiency ultimately decreased mRNA expression of Mcl‐1 [11], thus, we initially hypothesized that DHFR may regulate the transcriptional activity of STAT3, thereby affecting Mcl‐1 transcription. However, contrary to our prediction, the expression of phosphorylated and total STAT3 did not fluctuate (data not shown), and Mcl‐1 mRNA expression remained relatively unchanged after PYR treatment (Figure 3C). This indicates that Mcl‐1 was not regulated at the transcriptional level. The stability of the Mcl‐1 protein was undermined when protein synthesis was inhibited by CHX, whereas Mcl‐1 protein degradation was reduced upon proteasome inhibitor treatment (Figure 3D,E). These results indicate that Mcl‐1 is regulated by posttranslational mechanisms, particularly through proteasome‐mediated decay, in the context of PYR treatment.

The regulation of the anti‐apoptotic activity of Mcl‐1 is mainly attributed to modulation of its levels, rather than changes in its activity [8]; thus, Mcl‐1 possesses distinct structural features, notably the PEST regions, which regulate protein degradation and are enriched in proline, glutamic acid, serine, and threonine residues. These highly adjustable regions set Mcl‐1 apart from other antiapoptotic proteins with sequential similarity, such as Bcl‐2, and enable it to exert more flexible activity in blocking apoptosis due to its rapid turnover [24]. Therefore, phosphorylation in the PEST region by various kinases, followed by ubiquitination or de‐ubiquitination, is one of the most important determinants of the fate of Mcl‐1 [25]. As mentioned above, Mcl‐1 is processed via the proteasome‐associated pathway after PYR treatment, and certain kinases that destabilize Mcl‐1 protein are strongly suspected to be involved. Diverse kinases are generally implicated in the phosphorylation of PEST regions that direct Mcl‐1 degradation, including GSK3, JNK, p38, ERK, CDK, and others based on the specific cellular context [26]. Further, several ubiquitin ligases participate in fine‐tuning this powerful anti‐apoptotic protein. Other E3 ubiquitin ligases associated with Mcl‐1 include MARCH5, Parkin, β‐TrCP, FBW7, and TRIM17, starting with Mule, the first identified ligase [17]. These kinases and ligases work in concert to regulate Mcl‐1 degradation; hence, identifying which kinase–ligase axis is disrupted by PYR will be valuable for a comprehensive understanding of its mechanism. However, we have not identified the precise mechanism or the mediators by which Mcl‐1 phosphorylation was promoted, resulting in its clearance after PYR administration in this study. Therefore, further investigation is warranted, including kinase screening and the upstream kinase validation using specific inhibitors, followed by E3 ligase identification through ubiquitination and coimmunoprecipitation assays.

The YD‐15 cell line was established from the high‐grade MEC of the tongue and demonstrated a limited ability to generate tumors in nude mice [27]. Due to this limitation, we were unable to conduct mouse xenograft experiments to assess the potential of PYR as a cancer therapeutic agent in vivo. Instead, we adopted a hanging drop 3D culture model, considering its well‐known ability to more accurately recapitulate in vivo biology compared with 2D culture settings. The data obtained from the 3D culture system provide valuable insights to partially compensate for the lack of in vivo xenograft experiments. The 3D culture model has been widely recognized for its reduced drug sensitivity due to its multilayered structural features compared with the 2D culture model [28]. Hence, the drug concentration needs to be increased when conducting experiments using the 3D culture model, with the optimal concentration being highly dependent on the cell and drug types. To identify the appropriate PYR concentration, we referred to several earlier studies that compared the IC_50_ values of several drugs across different cell lines in both 2D and 3D culture models. One study reported differences in the IC_50_ values of clinical drugs across multiple colorectal cancer cell lines ranging from a 1.7‐ to 5.5‐fold change [29]. Alwahsh et al. measured IC_50_ changes using various cell lines, and the IC₅₀ values increased by as little as 1.35‐fold and up to 4.92‐fold in 3D cultures compared with 2D cultures [30]. Further, human breast cancer MCF‐7 cells require approximately three times higher concentrations of arsenic disulfide to induce cell death in 3D cultures compared with 2D culture models [31]. The IC_50_ values of doxorubicin, 5‐FU, and cisplatin in several colorectal cancer cell lines increased in 3D cultures compared with 2D cultures, ranging from 1.2‐ to 10‐fold based on the drug and cell line, with a few decreased or unchanged responses [32]. In our study, we treated spheroids with 120 μM of PYR, a fourfold increase in concentration compared with its 2D counterpart, which falls within the range reported in the references, and readily observed the apoptotic effect of PYR (Figure 5). We did not measure the IC₅₀ shift of PYR specifically in YD‐15 cells; however, the use of a fourfold higher concentration in our 3D model is consistent with the fold changes reported in these studies and is thus considered appropriate for assessing PYR efficacy. Furthermore, our 3D model provides useful preclinical insights, but further animal studies are warranted to clarify the in vivo efficacy of PYR. We remain optimistic about its therapeutic potential, considering that a growing body of literature has reported the capability of PYR to reduce tumor burden in mouse models with multiple tumor backgrounds [33, 34, 35].

The pharmacokinetics of a drug are an important aspect of its clinical translatability. We were unable to assess the overall plasma concentration due to the lack of in vivo experiments; however, several studies have provided evidence of the biocompatibility and pharmacokinetic behavior of PYR. One study revealed that the average plasma concentration was approximately 1.78 μg/mL over 24 h in 11 patients positive for human immunodeficiency virus receiving 50 mg of PYR daily for 3 weeks [36]. Using a microbiological method, Weidekamm et al. reported a plasma concentration of 0.796 μg/mL in 14 volunteers after a single tablet administration [37]. In another study, 12 healthy young volunteers received a 50‐mg dose of PYR, resulting in a plasma concentration of 2.37 μg/mL [38]. Further, intravenous PYR administration at 1 mg/kg over 2 h resulted in a peak plasma concentration of approximately 2089 ng/mL in healthy volunteers [39]. These residual plasma concentrations are significantly lower than the effective concentrations used in our in vitro experiments. Therefore, alternative approaches, such as drug modification or nanoparticle‐based delivery systems, may be considered. Several studies have revealed that PYR derivatives, including methylbenzoprim, WCDD115, and other optimized analogs, demonstrate improved anticancer efficacy, such as cell cycle arrest, NRF2 suppression, and high‐affinity DHFR inhibition, at significantly lower concentrations than the parent compound [40, 41, 42]. Further, various nanoparticle‐based delivery systems, such as mPEG–PCL nanoparticles, carbon nanotube carriers, and poloxamer 407 micelles, have been developed to improve the solubility, bioavailability, and sustained release of PYR while minimizing systemic toxicity [43, 44, 45].

In conclusion, for the first time, we revealed that the FDA‐approved antiparasitic drug PYR can restrain the growth of human MEC as well as kill parasites, which can be attributed to the concurrent induction of extrinsic and intrinsic apoptosis through caspase 8 activation and Mcl‐1 protein destabilization, respectively. These results indicate that PYR can be repurposed as an anticancer drug for MEC in a clinical setting.

## Author Contributions

**Hyun‐Ji Kim:** conceptualization, formal analysis, validation, visualization, and writing – original draft. **Dong‐Guk Park and Su‐Jung Choi:** investigation and methodology. **Jae‐Jin Cho and Seong‐Doo Hong:** writing – review; **Sung‐Dae Cho:** supervision and writing – review and editing.

## Ethics Statement

The authors have nothing to report.

## Consent

The authors have nothing to report.

## Conflicts of Interest

The authors declare no conflicts of interest.

## Peer Review

The peer review history for this article is available at https://www.webofscience.com/api/gateway/wos/peer‐review/10.1111/jop.70024.

## Supporting information


**Data S1:** Supporting Information.
**Figure S1:** The effect of PYR on DR5 expression. YD‐15 was exposed to DMSO or the designated concentrations of PYR for 48 h. The expression of DR5 is shown in the western blot images with β‐actin serving as a loading control.
**Figure S2:** The effect of PYR on pro‐ and antiapoptotic proteins. YD‐15 was exposed to DMSO or the designated concentrations of PYR for 48 h. (A) The expression of Bak, Bax, Bim, Bid, Bad, Bcl‐2, and Bcl‐XL is shown in the western blot images with β‐actin serving as a loading control.
**Figure S3:** Mcl‐1 overexpression. YD‐15 cell was transfected with blank pcDNA3.1 vector or the pcDNA3.1‐Mcl‐1 vector for 12 h and followed by PYR treatment for 48 h. The expression of Mcl‐1 is shown in the western blot images with β‐actin serving as a loading control.
**Figure S4:** Dose‐dependent cytotoxic effect of PYR on YD‐15 3D spheroids. YD‐15 cell spheroids were exposed to DMSO or the indicated concentrations of PYR for 48 h. The images show live cells stained with Calcein AM (green) and dead cells stained with EthD‐1 (red). Magnification, ×40; Scale bar, 100 μm.
**Table S1:** The information of antibodies and dilution conditions.

## Data Availability

The data that supports the findings of this study are available within this article and its Supporting Information.
