# Supplementary material for: Pyrimethamine Triggers the Apoptotic Pathway in Mucoepidermoid Carcinoma in Cell‐Based Models
Source: J Oral Pathol Med. 2025 Aug 6;54(8):723–32. doi: 10.1111/jop.70024 (PMC12419981; doi:10.1111/jop.70024)
Supplement: Supplementary file 1 — Data S1: Supporting Information. Figure S1: The effect of PYR on DR5 expression. YD‐15 was exposed to DMSO or the designated concentrations of PYR for 48 h. The expression of DR5 is shown in the western blot images with β‐actin serving as a loading control. Figure S2: The effect of PYR on pro‐ and antiapoptotic proteins. YD‐15 was exposed to DMSO or the designated concentrations of PYR for 48 h. (A) The expression of Bak, Bax, Bim, Bid, Bad, Bcl‐2, and Bcl‐XL is shown in the western blot images with β‐actin serving as a loading control. Figure S3: Mcl‐1 overexpression. YD‐15 cell was transfected with blank pcDNA3.1 vector or the pcDNA3.1‐Mcl‐1 vector for 12 h and followed by PYR treatment for 48 h. The expression of Mcl‐1 is shown in the western blot images with β‐actin serving as a loading control. Figure S4: Dose‐dependent cytotoxic effect of PYR on YD‐15 3D spheroids. YD‐15 cell spheroids were exposed to DMSO or the indicated concentrations of PYR for 48 h. The images show live cells stained with Calcein AM (green) and dead cells stained with EthD‐1 (red). Magnification, ×40; Scale bar, 100 μm. Table S1: The information of antibodies and dilution conditions. [file JOP-54-723-s001.docx]

**Supporting Information**

**Pyrimethamine triggers the apoptotic pathway in mucoepidermoid carcinoma in cell-based models**

Hyun-Ji Kim^1^, Dong-Guk Park^1^, Su-Jung Choi^1^, Jae-Jin Cho^2^, Seong-Doo Hong^1^, and Sung-Dae Cho^1,3*^

^1^Department of Oral Pathology, School of Dentistry and Dental Research Institute, Seoul National University, Seoul 03080, Republic of Korea

^2^Department of Dental Regenerative Biotechnology, School of Dentistry, Seoul 03080, Republic of Korea

^3^Center of Excellence in Genomics and Precision Dentistry, Department of Physiology, Faculty of Dentistry, Chulalongkorn University, Bangkok 10330, Thailand

^*^, Corresponding author;

**Sung-Dae Cho** Department of Oral Pathology, School of Dentistry and Dental Research Institute, Seoul National University, Seoul, 03080, Republic of Korea. Tel: 82-2-740-8647, E-mail: [efiwdsc@snu.ac.kr](file:///C:\Users\SNU\Desktop\Oridonin_MEC%20논문%20작성중\Molecules\efiwdsc@snu.ac.kr)

**Supplementary materials and methods**

*Cell counting kit-8 assay*

Following the manufacturer’s instructions, 10 µl of CCK-8 solution (Dojindo Laboratories, Kumamoto, Japan) was carefully added to cells cultured in 96-well plates and treated with the specified concentrations of PYR for 48 h. After incubating for 2-3 h, the absorbance in each well was measured at 482 nm using a Chameleon microplate reader (Hidex, Turku, Finland).

*Trypan blue assay*

Cells were mixed with 0.4% trypan blue solution (Lonza, USA) in a 1:1 ratio, and cell viability was automatically assessed using a CytoSMART cell counter (Corning, Tewksbury, MA, USA).

*Live & Dead assay*

Cells were stained with 4 μM EthD-1 and 2 μM Calcein-AM, both diluted in phosphate-buffered saline and incubated for 30 min (2D cultures) or 4 h (3D cultures) at RT. Fluorescent images were captured using a fluorescence microscope (Leica DM5000B; Leica Microsystems GmbH, Wetzlar, Hesse, Germany).

*Assessment of nuclear morphology alterations*

ells pre-fixed with 70% ethanol at −20°C overnight were mounted on glass slides and stained with 2 μg/mL 4',6-diamidino-2-phenylindole solution for 10 minutes. Apoptotic nuclear changes were subsequently captured using a fluorescence microscope (Leica DMi8; Leica Microsystems GmbH, Wetzlar, Hesse, Germany).

*Cell cycle distribution measurement*

Cells were fixed with 70% ethanol at −20°C overnight and subsequently stained with 20 µg/mL propidium iodide solution (Sigma, St. Louis, MO, USA) containing 20 µg/mL RNase A (Thermo Scientific., Waltham, MA, USA) for 15 min in a 37°C incubator. Cell cycle distribution was analyzed using an LSR Fortessa X-20 (BD Biosciences, San Jose, CA, USA) and further analyzed with FlowJo software, version 10 (FlowJo LLC, Ashland, OR, USA).

*Apoptotic cell population assessment*

According to the manufacturer’s manual, cells were stained with a FITC Annexin V Apoptosis Detection Kit (BD Pharmingen, San Jose, CA, USA). The Annexin V-positive population was evaluated using an LSR Fortessa X-20 (BD Biosciences, San Jose, CA, USA), and further analyzed with FlowJo software, version 10 (FlowJo LLC, Ashland, OR, USA).

**Supplementary Figure 1. The effect of PYR on DR5 expression.** YD-15 was exposed to DMSO or the designated concentrations of PYR for 48 h. The expression of DR5 is shown in the western blot images with β-actin serving as a loading control.


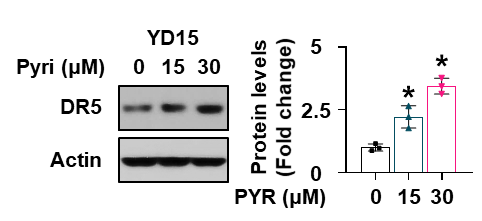


**Supplementary Figure 2. The effect of PYR on pro- and anti-apoptotic proteins.** YD-15 was exposed to DMSO or the designated concentrations of PYR for 48 h. (A) The expression of Bak, Bax, Bim, Bid, Bad, Bcl-2, and Bcl-XL is shown in the western blot images with β-actin serving as a loading control.


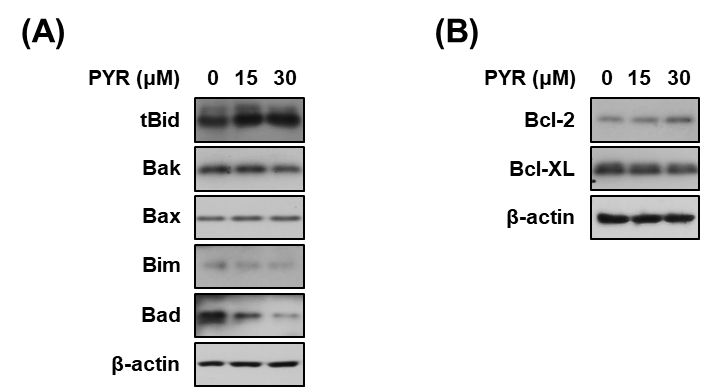


**Supplementary Figure 3. Mcl-1 overexpression.** YD-15 cell was transfected with blank pcDNA3.1 vector or the pcDNA3.1-Mcl-1 vector for 12 h and followed by PYR treatment for 48 h. The expression of Mcl-1 is shown in the western blot images with β-actin serving as a loading control.


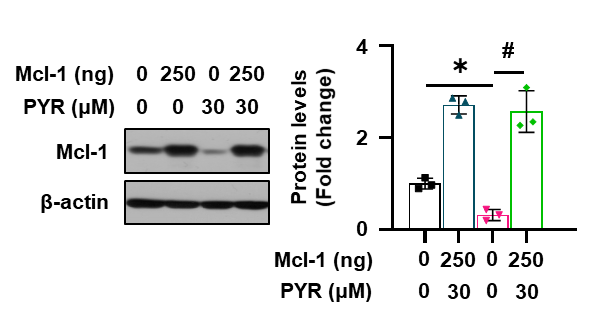


**Supplementary Figure 4. Dose-dependent cytotoxic effect of PYR on YD-15 3D spheroids.** YD-15 cell spheroids were exposed to DMSO or the indicated concentrations of PYR for 48 h. The images show live cells stained with Calcein AM (green) and dead cells stained with EthD-1 (red). Magnification, ×40; Scale bar, 100μm.


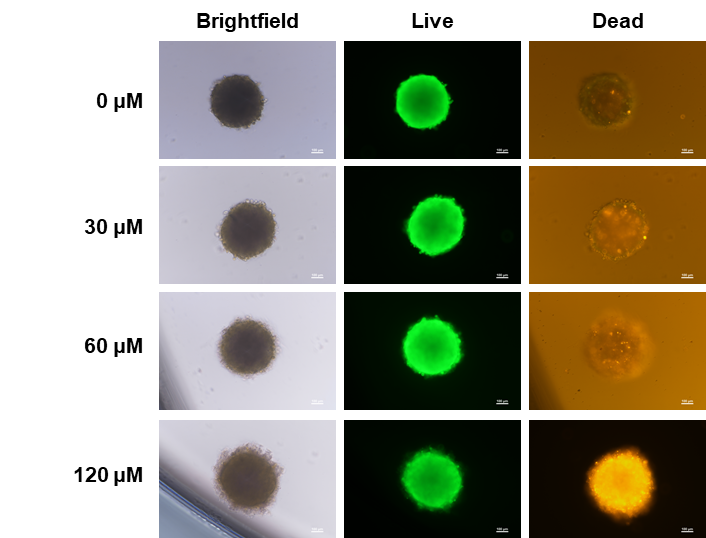


**Supplementary table 1. The information of antibodies and dilution conditions.**

| **Antibody name** | **Supplier and catalog number** | | **1^st^ dilution condition** | **2^nd^ dilution condition** |
| --- | --- | --- | --- | --- |
| **c-PARP** | Cell signaling | 9541 | 1:1000 | 1:2000 |
| **c-caspase 3** | Cell signaling | 9664 | 1:1000 | 1:2000 |
| **c-caspase 8** | Cell signaling | 9496 | 1:1000 | 1:2000 |
| **c-caspase 9** | Cell signaling | 9501 | 1:1000 | 1:2000 |
| **MCL-1** | Cell signaling | 5453 | 1:1000 | 1:2000 |
| **Bid** | Cell signaling | 2002 | 1:1000 | 1:2000 |
| **Bak** | Cell signaling | 3814 | 1:1000 | 1:2000 |
| **Bax** | Cell signaling | 2772 | 1:1000 | 1:2000 |
| **Bim** | Cell signaling | 2933 | 1:1000 | 1:2000 |
| **Bid** | Cell signaling | 2002 | 1:1000 | 1:2000 |
| **Bad** | Cell signaling | 9292 | 1:1000 | 1:2000 |
| **Bcl-2** | Cell signaling | 4223 | 1:1000 | 1:2000 |
| **Bcl-xL** | Cell signaling | 2764 | 1:1000 | 1:2000 |
| **β-actin** | SANTA CRUZ BIOTECH | sc-47778 | 1:3000 | 1:4000 |
